# Extra-articular migration of PEEK interference screw after anterior cruciate ligament reconstruction: a report of two cases

**DOI:** 10.1186/s12891-021-04387-2

**Published:** 2021-05-29

**Authors:** Chao-Hua Fang, Ming Li, Yun-Feng Zhang, Hua Liu

**Affiliations:** 1 Department of Joint Surgery, Ningbo No.6 Hospital, No.1059 Zhongshan road, Yinzhou District, Zhejiang 315000 Ningbo, People’s Republic of China; 2 School of Biomedical Engineering, Shanghai Jiao Tong University, No.1954 Huashan Road, Xuhui District, 200240 Shanghai, People’s Republic of China

**Keywords:** Polyetheretherketone, PEEK, Anterior cruciate ligament, Interference screw, Migration, Case report

## Abstract

**Background:**

The interference screw is the most popular device that fixes the graft for anterior cruciate ligament reconstruction, reducing the incidence of windshield effect and bungee effect. For the screw, either metallic, “bioresorbable,” or polyetheretherketone (PEEK) material is available. PEEK is popular and extensively used due to its stability, biocompatibility, radiolucency, and elastic modulus. Rare relevant complications were reported, but here, we report two cases of extra-articular migrations of PEEK interference screw from the tibial tunnel after anterior cruciate reconstruction.

**Case report:**

An 18-year-old boy and a 56-year-old woman underwent anterior cruciate ligament reconstruction using a PEEK interference screw to fix the graft in the tibial tunnel. They suffered from screw extrusion from the tibial tunnel after 40 days and six months, respectively, with an incision rupture or palpable subcutaneous mass. They underwent a second operation and recovered well.

**Conclusions:**

The exact incidence of extra-articular migrations of PEEK interference screws is unknown, but it seems to be quite low; despite this and its uncertain cause, the negative effects caused by the PEEK material need to be considered.

## Introduction

A secure fixation of the graft in the femoral and tibial tunnels is the prerequisite of a successful anterior cruciate ligament (ACL) reconstruction. Interference screws can be used either for soft-tissue grafts or bone–patellar tendon–bone grafts to reduce the incidence of windshield effect and bungee effect, and it has been shown to be a highly successful and reproducible technique with excellent long-term outcomes [1]. Regarding the materials for interference screws, titanium screws can lead to damage on the graft during screw insertion because of its hardness and artifacts on magnetic resonance imaging (MRI) for its metallic properties. “Bioresorbable” interference screws, mostly made from poly-L-lactic acid (PLLA) or poly-glycolic acid organic polymers, may lead to bone destruction and cyst formation during the hydrolytic process [2]. In addition, pretibial pseudocyst and pain at the tibial screw site were reported as complications [3, 4]. Another option is the polyetheretherketone (PEEK) screws [5]. PEEK is one of the polyaryletherketone (PAEK) polymers used as implants after the approval by the US Food and Drug administration in 1990 s [6]. Because of its compatibility, radiolucency, and elastic modulus, it becomes popular and is used extensively in trauma, joint and spinal implants [7–10]. The PEEK interference screws used in ACL reconstruction have excellent outcomes. Compared to TransFix (Arthrex, Inc, Naples, Florida) and EndoButton (Smith & Nephew Endoscopy, Mansfield, Massachusetts), PEEK interference screws do not have an effect on tunnel widening after hamstring ACL reconstruction [11]. Satisfactory clinical results were achieved when compared with titanium interference screws or cross-pin fixation in arthroscopic ACL reconstruction [5, 12].

Here, we present two cases of extra-articular migration of PEEK interference screws from the tibial tunnel, with or without wound rupture, at 40 days and 12 months after ACL reconstruction, respectively. To our knowledge, this is the first case report of this complication of commonly used PEEK screws.

## Case reports

### Case 1

**Fig. 1:**
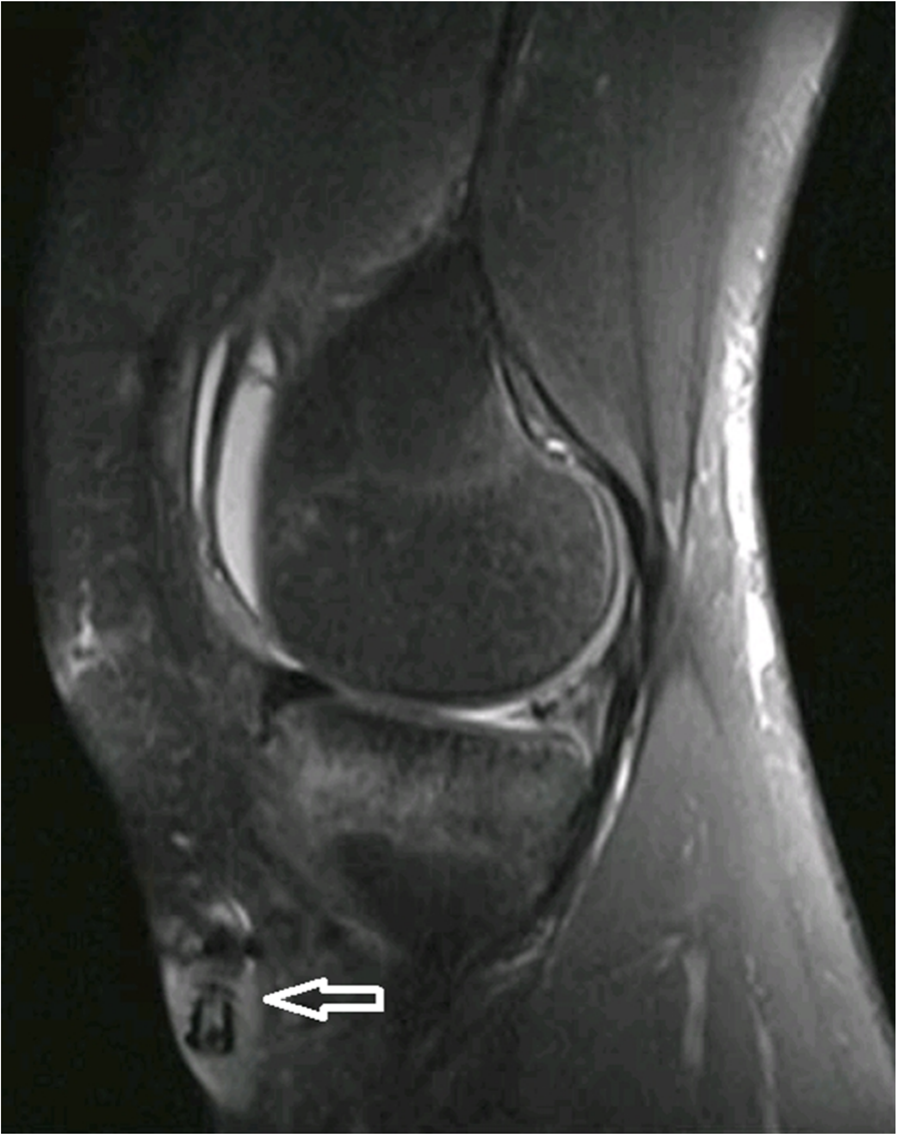
The white arrow show the interference screw extrude out from the tibial tunnel totally

An 18-year-old boy underwent ACL reconstruction with an autograft of quadruple hamstring and anterior half of the peroneus longus tendon of his left knee after a twisting sports injury six months prior to surgery. The diameter of the autograft was 7 mm, which was suspended with an Endobutton (Smith & Nephew, Memphis, TN, USA) at the femoral tunnel. An 8 mm × 25 mm PEEK interference screw (Smith & Nephew, Memphis, TN, USA) was used to fix the graft in the 7-mm tibial tunnel. The patient recovered well and was discharged two days post-operation. His wound healed well after two weeks, so the sutures were removed in an outpatient department, and knee flexion was encouraged. Forty days after the operation, the patient came back and complained of tibial wound rupture for one day after a 1-week history of swelling and pain over the anterior aspect of the proximal tibia. Knee examination showed a 1-cm rupture in the tibial wound without obvious redness and effusion, but with focal soft-tissue swelling and moderate tenderness around the wound. The knee joint also had no obvious swelling. Laboratory evaluation revealed no systemic signs of infection, with an ESR of 35 mm/h, CRP of 2.9 mg/L, and WBC of 6.9 × 10^9^/L. The knee joint fluid evaluation revealed no signs of infection, with a nucleated cell count of 900 × 10^6^/L. The joint fluid smear of bacterium and fungus was negative. The MRI scan revealed the intact screw extrusion from the tibial tunnel to subcutaneous tissue (Fig. 1).

Surgery was performed to remove the screw. When the wound rupture was incised and enlarged, the screw was found in the subcutaneous tissue surrounded by a small amount of clear fluid. The whole screw was removed, and its surrounding soft tissue was fetched for germiculture. After the debridement of the adjacent soft-tissue and tibial tunnel, sufficient irrigation was performed, and a vacuum sealing drainage dressing (VSD) covered the wound with a continuous negative pressure suction to promote healing.

**Fig. 2:**
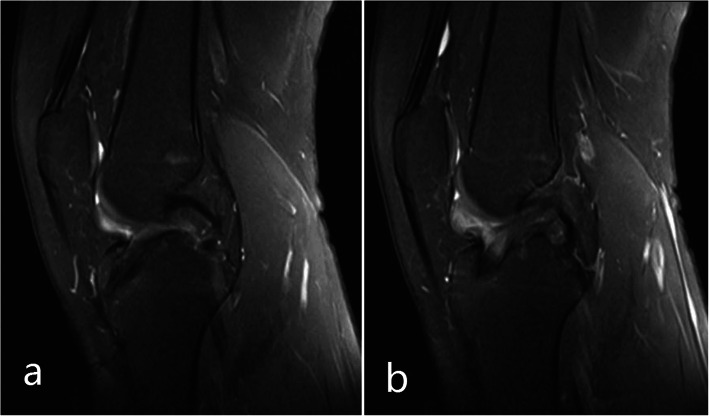
**a** and **b** show the tibial tunnel and regenerated anterior cruciate ligament 2 years after the screw was removed

VSD was removed after three days, and wound dressing was performed every three days. The cultures of preoperative joint fluid and intraoperative soft tissue were both negative. Therefore, he was discharged from the hospital with a smoothly healed wound two weeks post-reoperation. After two years, he recovered uneventfully, with a knee joint range of motion of 120° of flexion to 0° of extension. The Lachman test was positive, but the pivot-shift test negative. He was able to return to simple sport activities, such as jogging, without continued symptoms. The MRI scan showed a continuously regenerating ACL (Fig. 2).

### Case 2

**Fig. 3:**
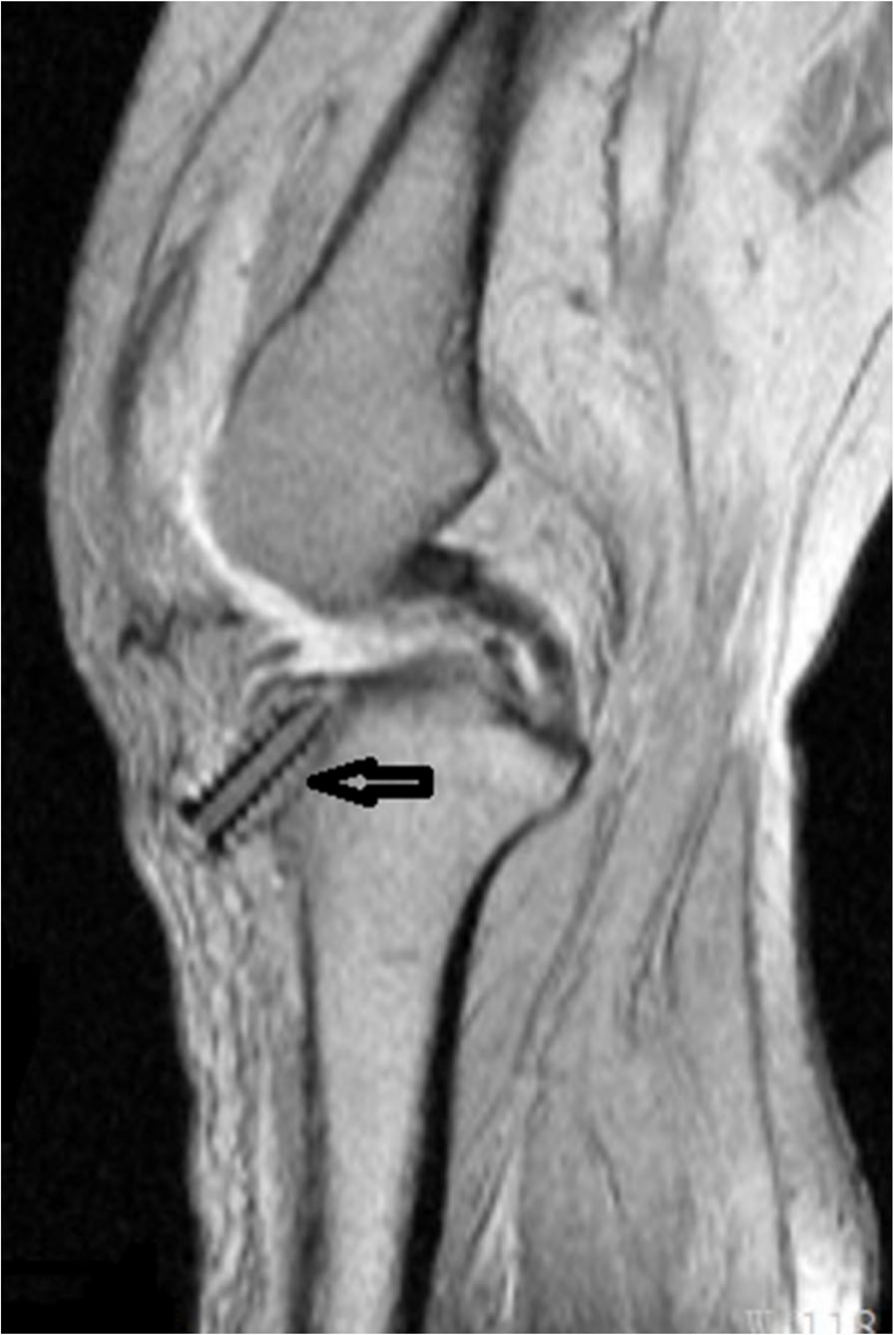
The black arrow show the interference screw extrude out partially

A 56-year-old woman underwent ACL reconstruction with an autograft of quadruple hamstring and allograft tendon of her left knee after a fall two months prior to surgery. The total diameter of the graft was 9 mm, and it was suspended with an Endobutton (Smith & Nephew, Memphis, TN, USA) at the femoral tunnel. A 9 mm × 25 mm PEEK interference screw (Smith & Nephew, Memphis, TN, USA) was used to fix the graft in the 9-mm tibial tunnel. The patient recovered well and was discharged two days post-operation. Her wound healed well, and the sutures were removed in an outpatient department. Knee flexion was encouraged two weeks post-operation. The patient regained the normal range of knee motion, but she complained of discomfort in the pretibial wound six months post-operation. Physical examination revealed a bulge under the wound scar, with a firm and immobile palpable mass. The MRI scan showed a part of screw extrusion from the tibial tunnel to the subcutaneous tissue (Fig. 3).

**Fig. 4:**
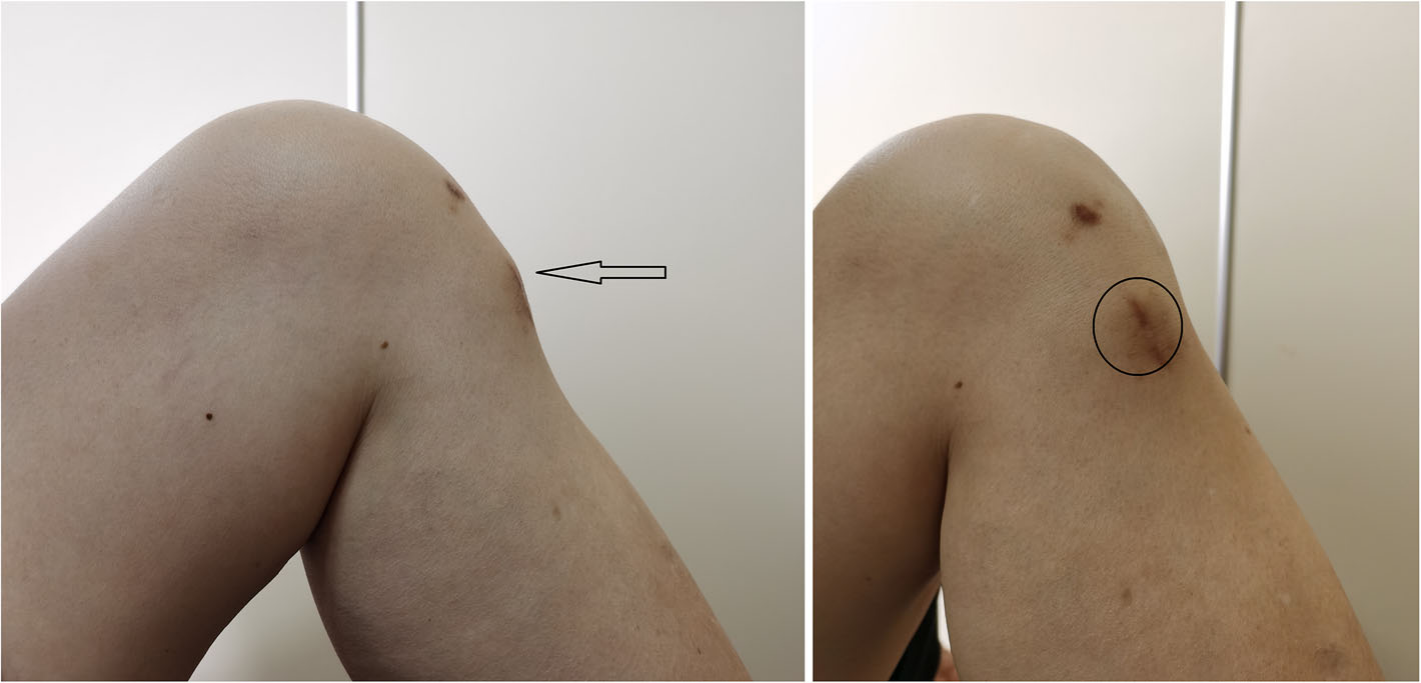
The black arrow and circle indicate subcutaneous buldge at lateral and front view respectively

**Fig. 5:**
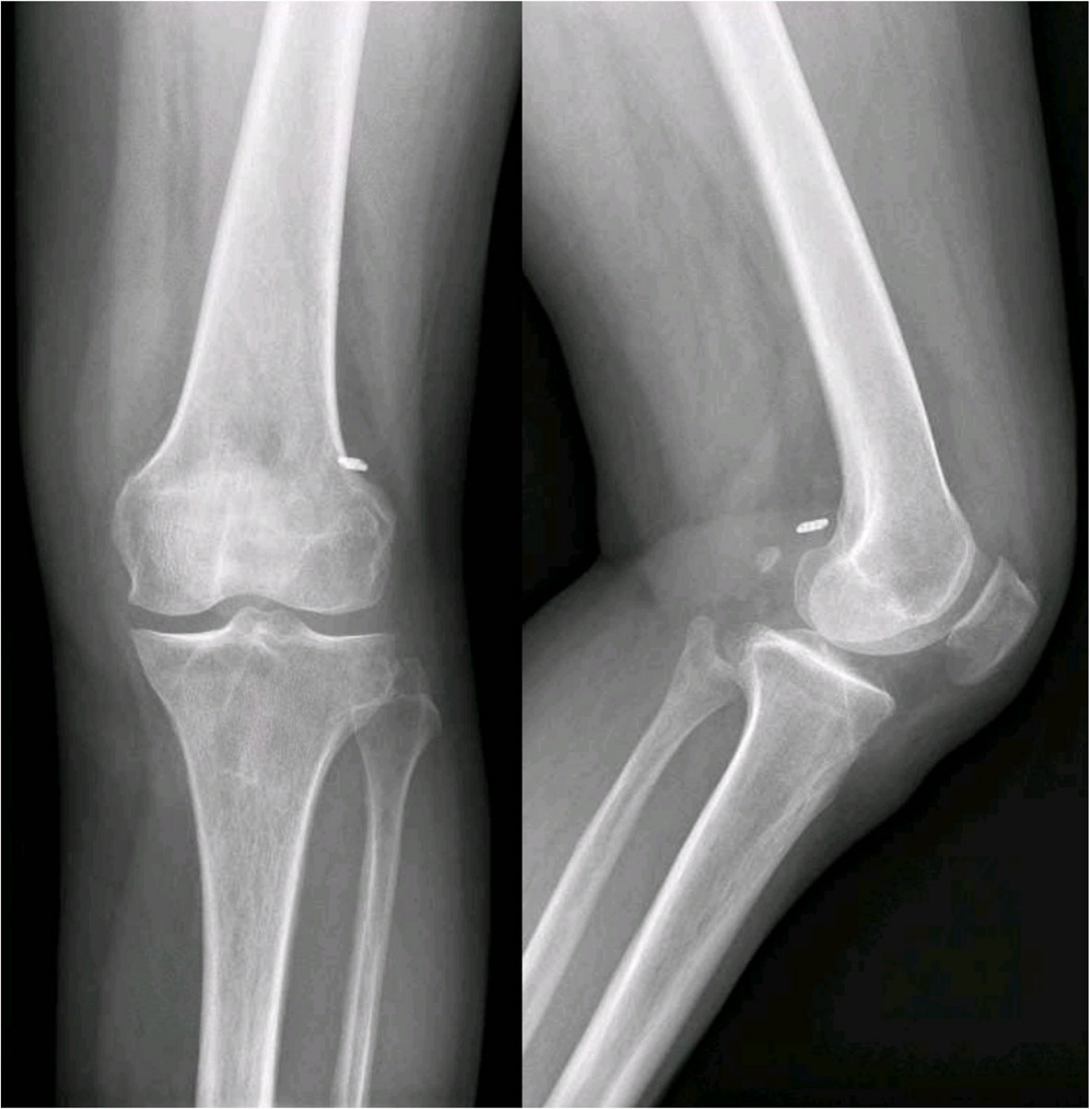
Front and side view of X ray manifest no evidence of enlargement of the tibial tunnel

**Fig. 6:**
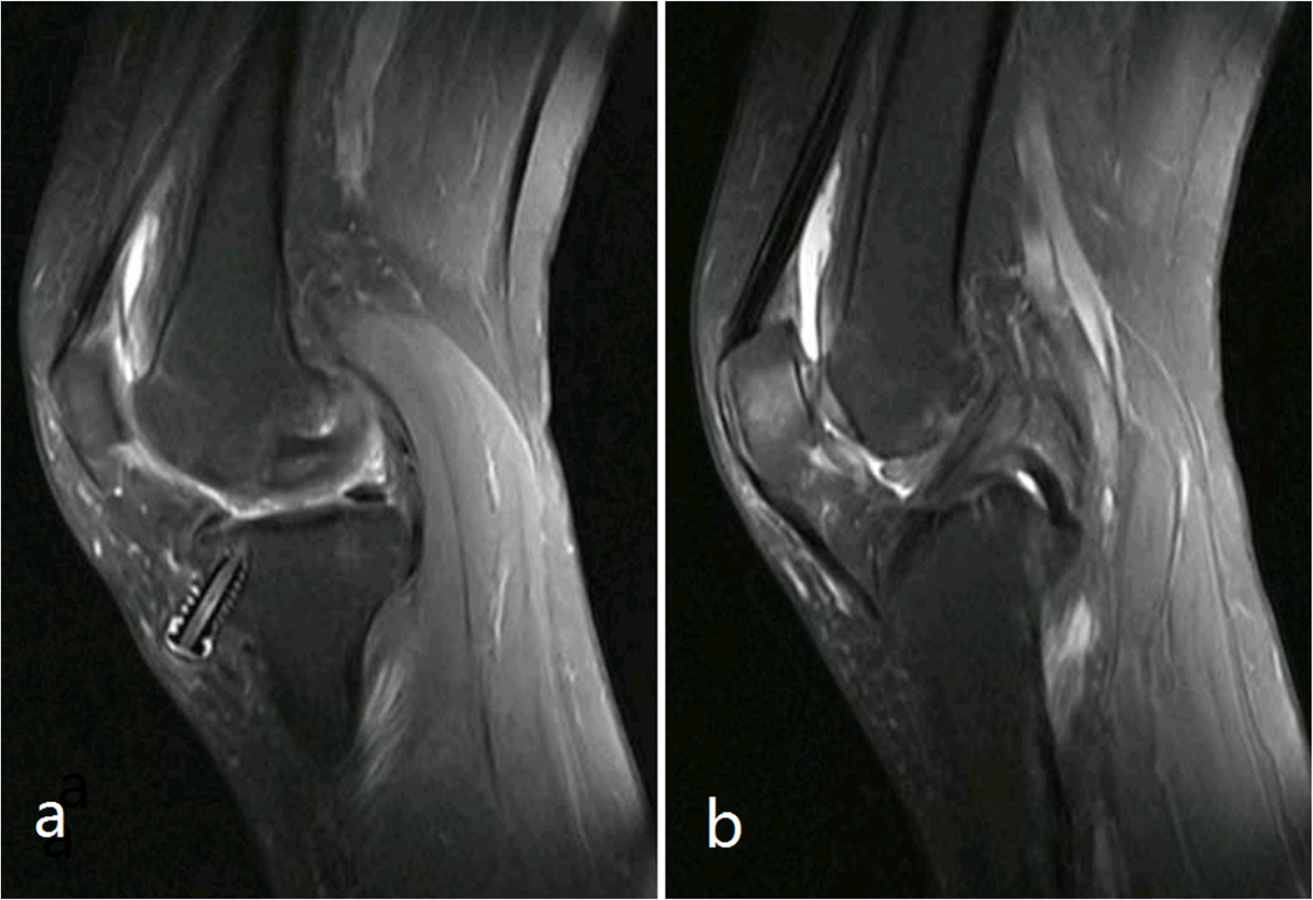
**a** show the screw extruded out even more and **b** show the continuous regenerated anterior cruciate ligament with good tension

**Fig. 7:**
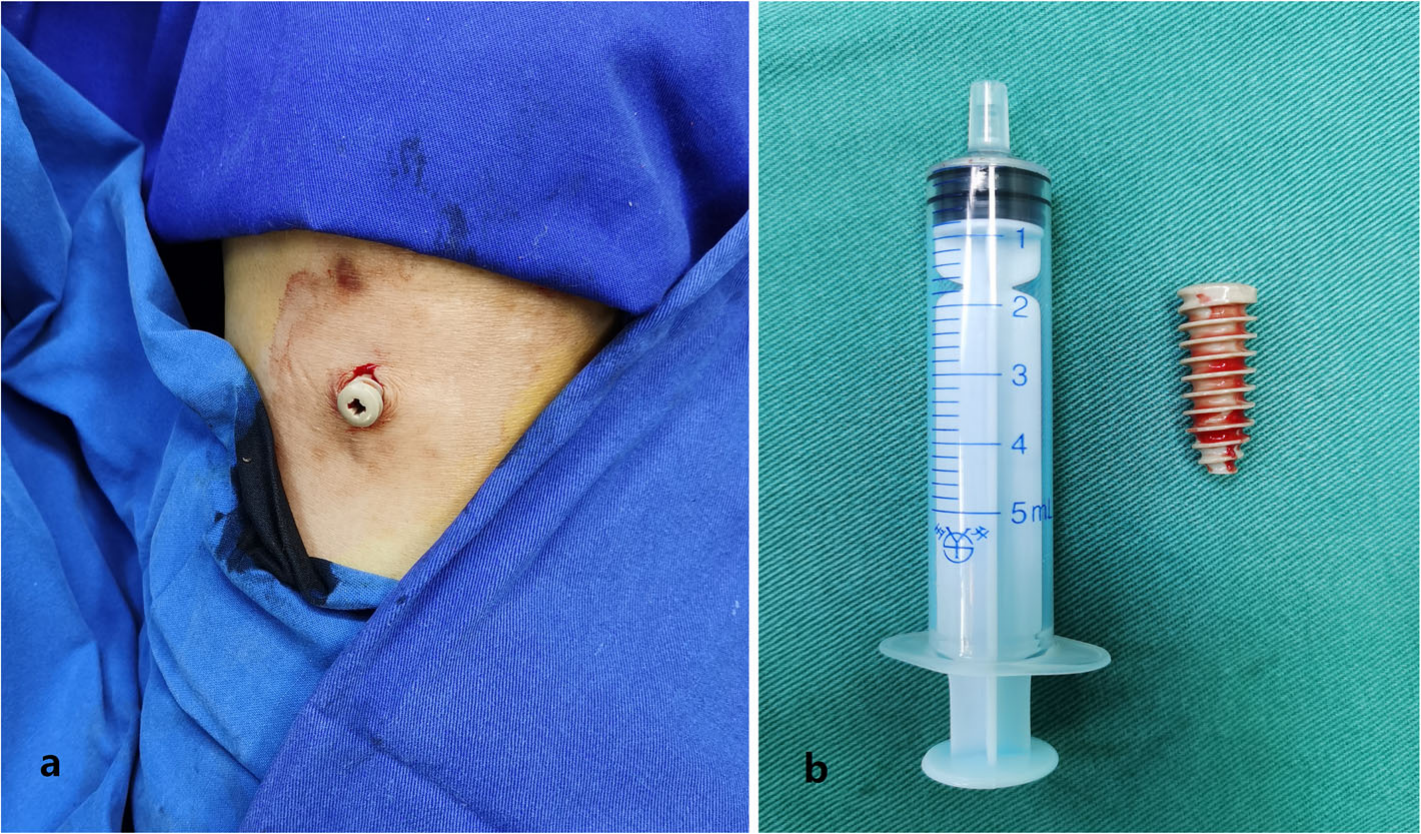
The interference screw was removed as a whole

However, she refused to undergo a second operation due to economic pressure and less obvious symptoms. After six months, the patient complained of increased subcutaneous bulge with tenderness (Fig. 4). The X-ray showed no tibial tunnel enlargement, while the MRI scan showed that the screw was extruded out even more (Figs. 5 and 6). Then, a second operation was performed to remove the whole screw successfully (Fig. 7). The patient recovered well and resumed her daily activities without any discomfort.

## Discussion

Interference screw migration is uncommon. Several cases of metallic interference screw migration have been reported in literatures, with femoral screws migrating into the joint cavity. All patients underwent ACL reconstruction with bone–patellar tendon–bone graft and received the reoperation after pulling out the femoral interference screw [13–18]. The causes of screw loosening can be multiple and includes size mismatch, screw divergence, poor bone quality, and bone resorption due to thermal necrosis caused by drilling or malfunctional remodeling phase of healing. Bone tunnel enlargement after ACL reconstructions could be another potential cause. During knee motion, the graft or regenerated ligament pulls the screw into the articular cavity. Among the reports retrieved in the literature, the most common material that migrates was PLLA, and the most common migration was to the intra-articular region rather than the extra-articular region [19, 20]. It also seems to be more frequent in the tibia. Besides the possible reasons mentioned above, the biological and chemical reactions occurring after the implantation of “bioresorbable” interference screws also need be considered [20].

To our knowledge, this is the first case report of PEEK interference screw migration after ACL reconstruction. Because a non-routine MRI scan was performed after the operation, a postoperative radiology of the interference screw with normal position was absent. However, we confirmed that the screw migrated after operation, because the well-trained surgeons (Dr Zhang and Dr Li) recruited the interference screws of proper size and made sure they had been inserted into the tibial tunnel completely with palpation during operation. However, unlike the metallic screw, which was pulled out of the femoral tunnel, the PEEK screw was extruded out of the tibial tunnel in these two cases. This is more similar with the cases of “bioresorbable” interference screw, which manifests as a palpable mass with or without wound dehiscence. The reason cannot be excluded as screw divergence, bone resorption, and tunnel enlargement resulting from windshield effect and bungee effect. The biological reactions of PEEK cannot also be neglected, with several cases recently reported in literatures. A rare case of allergic reaction seven days following PEEK cranioplasty was reported, with light yellow and transparent epidural effusion at the bilateral PEEK implant site. This reaction was successfully treated with dexamethasone and drainage [10]. One case of a pre-known severe type IV allergy to epoxy resin was reported with constant, severe pain in the shoulder area after rotator cuff reattachment surgery with PEEK implant. The patient had to underwent a reoperation to remove the implant, and a wire cerclage was used to fix the tendons. After the revision surgery, the patient fully recovered and was symptom-free [21]. Another case developed chronic systemic allergy with itching, erythema, periorbital swelling, and macroglossia after spine surgery with a PEEK implant. With the implant removed, these allergic symptoms completely resolved [22]. A case of articulation-related failures was reported after a Motec total wrist arthroplasty caused by an adverse reaction to PEEK particles in the surrounding synovia, resulting in revision surgery [23]. In addition to allergic and foreign body reaction, PEEK may exert a negative effect of integration with the host bone [24]. When mesenchymal cells are cultured on a PEEK material in vitro, a high level of interleukin-1β (IL-1β) is expressed, which is a marker of fibrous tissue formation. However, none of the bone formation markers, such as alkaline phosphatase or osteocalcin, were expressed. Significantly higher levels of cell necrosis, DNA damage, and apoptosis are also introduced [25, 26]. In addition, PEEK induces a stronger inflammatory response from peripheral blood mononuclear cells compared with titanium-6 aluminum-4 vanadium [27]. The negative results of in vivo studies are consistent with these in vitro studies. It has been demonstrated that PEEK cages tend to prevent bone integration, surrounded by fibrous connective tissue, and lead to nonunion [28]. PEEK particles caused a significantly higher peri-implant osteolysis than highly cross-linked polyethylene (HXLPE) particles [29]. In addition, carbon fiber-reinforced PEEK particles injected into the knee joints may induce a higher level of inflammatory cytokine expression (IL-1β, IL-6, and TNF-α) compared with ultra-high-molecular-weight polyethylene (UHMWPE) particles [30]. Given these negative systematic and osteocyte effects, the migration of PEEK interference screws may be more interpretable. Although the two patients suffered from screw migration, the course of migration differed significantly. In the first patient, a young boy, the whole screw was totally extruded out of the tunnel within 40 days, accompanied with wound dehiscence. In contrast, the second patient, an elderly woman, suffered from partial screw extrusion gradually. This can be explained by the difference in their age. The biological reactions to PEEK interference screws might be stronger at a young age. Similar to inflammation and bone reaction in a murine model exposed to polyethylene particles, the young mice group demonstrated a prompt inflammatory reaction, followed by a decrease in fully mineralized bone and subsequent increase in regenerating bone [31]. However, the cause of complication in these two cases is still uncertain. More studies need to be performed by material scientists to clarify these, focusing on the effect of PEEK material on organic bone tissues.

## Conclusions

The exact incidence of extra-articular migrations of PEEK interference screws is unknown, but it seems to be quite low; despite this and its uncertain cause, the negative effects caused by the PEEK material need to be considered.

## Data Availability

The datasets used and/or analysed during the current study are available from the corresponding author on reasonable request.

## Abbreviations

PEEK: Polyetheretherketone
ACL: Anterior cruciate ligament
PLLA: Poly-L-lactic acid
ESR: Erythrocyte Sedimentation Rate
CRP: C-reactive protein
WBC: White Blood Cell Count
MRI: Magnetic resonance imaging
VSD: Vacuum sealing drainage dressing
PAEKs: Polyaryletherketones
US: United states
DNA: Deoxyribonucleic acid
IL-1β: Interleukin-1β
HXLPE: Highly cross-linked polyethylene
UHMWPE: Ultra-high-molecular-weight-polyethylene
IL-6: Interleukin-6
TNF-α: Tumor necrosis factor-α

## References

[1] Bourke HE, Gordon DJ, Salmon LJ, Waller A, Linklater J, Pinczewski LA (2012). The outcome at 15 years of endoscopic anterior cruciate ligament reconstruction using hamstring tendon autograft for ‘‘isolated’’ anterior cruciate ligament rupture. J Bone Joint Surg Br.

[2] Chevallier R, Klouche S, Gerometta A, Bohu Y, Herman S, Lefevre N (2019). Bioabsorbable screws, whatever the composition, can result in symptomatic intra-osseous tibial tunnel cysts after ACL reconstruction. Knee Surg Sports Traumatol Arthrosc.

[3] Christodoulidis A, Barbareschi M, Molinari M (2018). A Delayed Formation of Pretibial Pseudocyst After Anterior Cruciate Reconstruction from Bioabsorbable Interference Screws: A Report of Two Cases and Short Review of the Literature. J Long Term Eff Med Implants.

[4] Kramer DE, Kalish LA, Kocher MS, Yen YM, Micheli LJ, Heyworth BE (2020). Complications of Bioabsorbable Tibial Interference Screws After Anterior Cruciate Ligament Reconstruction in Pediatric and Adolescent Athletes. Orthop J Sports Med.

[5] Shumborski S, Heath E, Salmon LJ, Roe JP, Pinczewski LA (2019). A randomized controlled trial of peek versus titanium interference screws for anterior cruciate ligament reconstruction with 2-year follow-up. Am J Sports Med.

[6] Kurtz SM, Devine JN (2007). PEEK biomaterials in trauma, orthopedic, and spinal implants. Biomaterials.

[7] Bartelstein MK, Van Citters DW, Weiser MC, Moucha CS (2017). Failure of a Polyaryletheretherketone-Cobalt-Chromium Composite Femoral Stem Due to Coating Separation and Subsidence: A Case Report. JBJS Case Connect.

[8] Hak DJ, Fader R, Baldini T, Chadayammuri VBS (2017). Locking screw-plate interface stability in carbon-fibre reinforced polyetheretherketone proximal humerus plates. Int Orthop.

[9] Krause KL, Obayashi JT, Bridges KJ, Raslan AM, Than KD (2018). Fivefold higher rate of pseudarthrosis with polyetheretherketone interbody device than with structural allograft used for 1-level anterior cervical discectomy and fusion. J Neurosurg Spine.

[10] Qiu S, You W, Wang H, Zhou X, Yang X (2019). Allergic Epidural Effusion Following Polyetheretherketone Cranioplasty. J Craniofac Surg.

[11] Uzumcugil O, Yalcinkaya M, Ozturkmen Y, Dikmen G, Caniklioglu M (2012). Effect of PEEK polymer on tunnel widening after hamstring ACL reconstruction. Orthopedics.

[12] Uzumcugil O, Dogan A, Dalyaman E, Yalcinkaya M, Akman E, Ozturkmen Y, Caniklioglu M (2010). AperFix versus transfix in reconstruction of anterior cruciate ligament. J Knee Surg.

[13] Hallett A, Mohammed A (2003). Displaced femoral interference screw causing locked knee. Injury.

[14] Moonot P, Allen P (2006). Intra-articular pull out of an interference screw after anterior cruciate ligament reconstruction. Knee Surg Sports Traumatol Arthrosc.

[15] Resinger C, Vécsei V, Heinz T, Nau T (2005). The removal of a dislocated femoral interference screw through a posteromedial portal. Arthroscopy.

[16] Bush-Joseph CA, Bach BR (1998). Migration of femoral interference screw after anterior cruciate ligament reconstruction. Am J Knee Surg.

[17] Karlakki SL, Downes ME (2003). Intra-articular migration of femoral interference screw: Open or arthroscopic removal. Arthroscopy.

[18] Sidhu DS, Wroble RR (1997). Intraarticular migration of a femoral interference fit screw. A complication of anterior cruciate ligament reconstruction. Am J Sports Med.

[19] Helito CP, Foni NO, Bonadio MB, Pécora JR, Demange MK, Angelini FJ (2017). Extra-articular and transcutaneous migration of the poly-l/d-lactide interference screw after popliteal tendon reconstruction. Rev Bras Ortop.

[20] Pereira H, Correlo VM, Silva-Correia J, Oliveira JM, Reis RL, Espregueira-Mendes J (2013). Migration of “bioabsorbable” screws in ACL repair. How much do we know? A systematic review. Knee Surg Sports Traumatol Arthrosc.

[21] Kofler L, Wambacher M, Schweinzer K, Scherl M, Kofler H (2017). Allergic Reaction to Polyether Ether Ketone Following Cross-Reactivity to Epoxy Resin. J Cutan Med Surg.

[22] Maldonado-Naranjo AL, Healy AT, Kalfas IH (2015). Polyetheretherketone (PEEK) intervertebral cage as a cause of chronic systemic allergy: a case report. Spine J.

[23] Karjalainen T, Pamilo K, Reito A (2018). Implant Failure After Motec Wrist Joint Prosthesis Due to Failure of Ball and Socket-Type Articulation-Two Patients With Adverse Reaction to Metal Debris and Polyether Ether Ketone. J Hand Surg Am.

[24] Shimizu T, Fujibayashi S, Yamaguchi S, Otsuki B, Matsuda S (2017). In vivo experimental study of anterior cervical fusion using bioactive polyetheretherketone in a canine model. Plos One.

[25] Olivares-Navarrete R, Gittens RA, Schneider JM, Hyzy SL, Haithcock DA, Ullrich PF, Schwartz Z, Boyan BD (2012). Osteoblasts exhibit a more differentiated phenotype and increased bone morphogenetic protein production on titanium alloy substrates than on polyether-ether-ketone. Spine J.

[26] Olivares-Navarrete R, Hyzy SL, Slosar PJ, Schneider JM, Schwartz Z, Boyan BD. Implant materials generate different peri-implant inflammatory factors: poly-ether-ether-ketone and promotes fibrosis and microtextured titanium promotes osteogenic factors. Spine. 2015;40(6):399–404. 10.1097/BRS.0000000000000778 PMC4363266 25584952

[27] Barkarmo S, Östberg AK, Johansson CB, Franco-Tabares S, Johansson PH, Dahlgren U, Stenport V (2018). Inflammatory cytokine release from human peripheral blood mononuclear cells exposed to polyetheretherketone and titanium-6 aluminum-4 vanadium in vitro. J Biomater Appl.

[28] Toth JM, Wang M, Estes BT, Scifert JL, Seim HB, Turner AS (2006). Polyetheretherketone as a biomaterial for spinal applications. Biomaterials.

[29] Du Z, Zhu Z, Wang Y (2018). The degree of peri-implant osteolysis induced by PEEK, CoCrMo, and HXLPE wear particles: a study based on a porous Ti6Al4V implant in a rabbit model. J Orthop Surg Res.

[30] Lorber V, Paulus AC, Buschmann A, Schmitt B, Grupp TM, Jansson V, Utzschneider S (2014). Elevated cytokine expression of different PEEK wear particles compared to UHMWPE in vivo. J Mater Sci Mater Med.

[31] Langlois J, Zaoui A, Bichara DA, Nich C, Bensidhoum M, Petite H, Muratoglu OK, Hamadouche M (2016). Biological reaction to polyethylene particles in a murine calvarial model is highly influenced by age. J Orthop Res.

